# A Monoclonal Antibody Targeting mGluR2 Inhibits H9 Subtype Influenza A Virus Infection In Vitro

**DOI:** 10.3390/vetsci13101001

**Published:** 2026-09-23

**Authors:** Pengfei Wang, Chuanchuan Xing, Shengmei Jiao, Jiantao Zhang, Jingfei Wang

**Affiliations:** 1 State Key Laboratory of Animal Disease Control and Prevention, Harbin Veterinary Research Institute, Chinese Academy of Agricultural Sciences, Harbin 150069, China; 2 College of Veterinary Medicine, Northeast Agricultural University, Harbin 150006, China

**Keywords:** mGluR2, influenza A virus, monoclonal antibodies, neutralization, host-directed therapy

## Abstract

Metabotropic glutamate receptor 2 (mGluR2) is a critical endocytic receptor for influenza A virus (IAV) entry, making it an attractive target for host-directed antiviral interventions. A recombinant truncated fragment of mGluR2 was expressed and purified from HEK293F cells in the present investigation. Immunization of BALB/c mice followed by splenocyte fusion yielded three stable hybridoma clones: 1G3B2, 2D5A9, and 4E1C6. All three monoclonal antibodies (mAbs) exhibited strong binding affinity to both denatured and native forms of mGluR2 in Western blot and immunofluorescence assays. In vitro neutralization assays revealed that clone 1G3B2 reduced H9 subtype IAV titers by approximately 7.7 folds and suppressed intracellular viral antigen expression in a dose-dependent manner. Clones 2D5A9 and 4E1C6, while showing no viral blocking efficacy, are valuable as research tools for Western blot and IFA applications. Genetic sequencing of 1G3B2 resolved its heavy and light chain variable regions, identifying a unique, prolonged heavy-chain CDR3 loop (TRHPVNNWAWFAY). This work reports three mAbs against mGluR2 and 1G3B2 could serve as a candidate agent for developing host-directed anti-influenza therapeutics.

## Simple Summary

Influenza A virus (IAV) exploits host cell receptors including metabotropic glutamate receptor 2 (mGluR2) for cellular entry and subsequent infection, rendering mGluR2 a promising target for host-directed antiviral intervention. In this study, we generated three novel monoclonal antibodies against mGluR2. Among these clones, 1G3B2, which harbours an extended heavy-chain CDR3 loop, exhibited in vitro inhibitory activity against H9-subtype IAV, markedly lowering viral titers and intracellular viral protein levels. This work describes three mAbs against mGluR2; of these, 1G3B2 serves both as a valuable detection reagent and a candidate antiviral agent.

## 1. Introduction

Metabotropic glutamate receptor 2 (mGluR2) is a canonical class C G-protein-coupled receptor (GPCR) that plays a crucial role in regulating neurotransmission in the mammalian central nervous system [1,2]. Structurally, mGluR2 features a large extracellular domain (ECD) with a unique Venus flytrap domain (VFT) that mediates ligand binding and receptor dimerization [3,4]. Its highly conserved structural features make it a promising target for host-directed therapeutic interventions [5]. Beyond its established neurological functions, recent studies have identified mGluR2 as a versatile host cell surface molecule. Notably, functional genomics screening has revealed a critical non-canonical role of mGluR2 as a key endocytic receptor for influenza A virus (IAV) entry [6]. During infection, mGluR2 mediates clathrin-mediated endocytosis (CME) of IAV, interacting with downstream host factors such as the calcium-activated potassium channel KCa1.1 and the tight junction protein Claudin-11 to facilitate viral internalization [7].

Influenza virus entry is initiated by the binding of the viral surface hemagglutinin (HA) protein to sialic acid receptors on the host cell surface [8]. Avian influenza viruses (AIVs) preferentially bind to α-2,3-linked sialic acid, while human influenza viruses favor the α-2,6 configuration [9,10]. However, recent studies have shown that sialic acid attachment alone is insufficient for viral particle internalization [11]. Multiple host co-receptors, including EGFR, VDCCs, and Nucleolin, must cooperate with sialic acid to activate downstream signaling pathways and mediate viral endocytosis [11]. This receptor-mediated endocytic mechanism is particularly critical for the pathogenesis of H9 subtype AIV, which has become endemic in poultry across at least 60 countries [12,13]. The H9N2 lineage poses a growing public health threat due to its potential for cross-species transmission to humans, with human seroprevalence reaching 10-37% [14,15,16].

Conventional antiviral strategies targeting viral surface glycoproteins often fail due to rapid viral antigenic drift [17]. In contrast, targeting conserved host endocytic receptors like mGluR2 provides a more resilient intervention strategy [5]. mGluR2-knockout mice exhibit significantly enhanced resistance to various influenza virus subtypes, and blocking mGluR2 effectively cuts off viral entry without inducing rapid drug resistance [6]. Monoclonal antibodies (mAbs) serve as indispensable tools to dissect functional domains of host receptors and can physically block virus–receptor interactions with high specificity [18]. However, well-characterized anti-mGluR2 mAbs that can efficiently block influenza endocytosis remain lacking [19].

In this study, we generated and evaluated a panel of anti-mGluR2 mAbs. We expressed recombinant truncated mGluR2 extracellular domain protein in mammalian HEK293F cells as an immunogen. Following hybridoma fusion, we successfully isolated three stable clones: 1G3B2, 2D5A9, and 4E1C6. Among them, clone 1G3B2 exhibited potent H9 virus-blocking efficacy and dose-dependent fluorescence inhibition. Conversely, clones 2D5A9 and 4E1C6 proved highly valuable for diagnostic assays such as Western blot and IFA. Furthermore, we precisely resolved the variable region gene rearrangements (VH/VL) and complementarity-determining region (CDR) sequences of 1G3B2. This work provides a mAb probe targeting mGluR2, offering a solid foundation for future structural biology research and humanization modifications of this antibody.

## 2. Materials and Methods

### 2.1. Cells, Virus, and Experimental Animals

HEK293F cells (Thermo Fisher Scientific, Waltham, MA, USA) were cultured at 37 °C in 293 Pro CD 293M medium (GenScript, Nanjing, China) with 5% CO_2_. SP2/0 cells (ATCC CRL-1581) were grown in DMEM medium (Gibco, Waltham, MA, USA) supplemented with 10% fetal bovine serum (FBS) at 37 °C with 5% CO_2_. HEK293T cells (ATCC CRL-3216) were maintained in DMEM medium (Gibco) supplemented with 5% FBS at 37 °C with 5% CO_2_. A549 cells (ATCC CCL-185) were cultured at 37 °C in Nutrient Mixture Kaighe’s Modification (F-12K Nut Mix, Gibco, Waltham, MA, USA) supplemented with 10% (*v*/*v*) FBS and 1% (*v*/*v*) penicillin–streptomycin with 5% CO_2_.

The H9N2 virus (CK/GX/S11583-NA staygold/2019) was propagated in 9-day-old specific-pathogen-free (SPF) embryonated chicken eggs. Viral titers were determined by the Reed-Muench method and expressed as 50% tissue culture infectious dose (TCID50) per mL.

The Laboratory Animal Center of Harbin Veterinary Research Institute provided the six-week-old female BALB/c mice used in our experiments. All animal experiments were conducted in accordance with the Institutional Ethics Committee of Harbin Veterinary Research Institute, the Chinese Academy of Agricultural Sciences (Approval No.:240813-01-GR).

### 2.2. Recombinant mGluR2 Protein Production

The genes encoding the extracellular domain of human mGluR2 (residues 1–493) (VFT, approximately residues 1–483) (approximately residues 484–493)) were cloned into the pcDNA3.4 vector with an 8× His tag at the C terminus. HEK293F cells (1 mL culture) were transfected with a mixture containing 2 μg of pcDNA3.4-mGluR2 plasmid and 4 μg of PEI at a cell density of 2 × 10^6^ cells/mL. The supernatant was collected 72 h post-transfection, centrifuged at 8000× *g* for 20 min, and purified using nickel affinity chromatography (Qiagen, Hilden, Germany). The eluted protein was concentrated and buffer-exchanged into 1× PBS buffer (pH 7.4) using an ultrafiltration spin column (Amicon Ultra-15, Merck Millipore, Billerica, MA, USA). Protein purity was confirmed by SDS-PAGE and Western blot analysis.

### 2.3. Production of Monoclonal Antibodies Against the mGluR2 Protein

To generate monoclonal antibodies against mGluR2, 6-week-old female BALB/c mice (*n* = 3) were immunized subcutaneously with 100 μg of eukaryotically expressed mGluR2 mixed with an equal volume of Complete Freund’s Adjuvant (CFA; Sigma-Aldrich, St. Louis, MO, USA). Three subsequent booster immunizations were administered at 14-day intervals using Incomplete Freund’s Adjuvant (Sigma-Aldrich, St. Louis, MO, USA). Prior to the fourth immunization, blood samples were collected via the tail vein to determine serum antibody titers. Three days following the final boost, splenocytes were harvested and fused with SP2/0 myeloma cells. The resulting hybridomas were cultured in Dulbecco’s Modified Eagle Medium (DMEM; Sigma-Aldrich) supplemented with 20% FBS and hypoxanthine-aminopterin-thymidine (HAT). Ten days post-fusion, culture supernatants were screened for anti-mGluR2 antibodies via indirect ELISA (iELISA). Positive clones were selected and subjected to three rounds of subcloning by limiting dilution. Stable monoclonal hybridoma cells were maintained in DMEM containing 20% FBS and 1% penicillin–streptomycin. The isotype of the monoclonal antibodies was determined using the SBA Clonotyping System-HRP kit (SouthernBiotech, Birmingham, AL, USA). The specificity of the antibodies toward the recombinant protein was validated using Western blotting and immunofluorescence assay (IFA).

### 2.4. SDS-PAGE and Western Blot

Loading buffer was added to the samples, which were then boiled for 10 min. Electrophoretic separation of proteins was performed on 10% polyacrylamide gels, followed by staining with Coomassie brilliant blue R-250 (Amresco, Solon, OH, USA). For Western blot assays, the separated proteins were subsequently transferred onto PVDF membranes (Thermo Fisher Scientific). The membranes were first blocked with 5% Difco skim milk for 2 h at 25 °C, and then reacted with a cocktail of primary antibodies (anti-His and anti-mGluR2) for 1 h. After four washes with TBST (0.5% Tween-20 in Tris-buffered saline), the membranes were exposed to IRDye 800CW goat anti-mouse IgG (H+L) secondary antibody (LI-COR, Lincoln, NE, USA, 1:5000) for another hour, then rinsed four additional times in TBST. The final immunoreactive bands were visualized by scanning on an Odyssey CLx Imaging System (LI-COR).

### 2.5. Enzyme-Linked Immunosorbent Assay (ELISA)

Purified mGluR2 protein was coated onto 96-well ELISA plates at a volume of 100 μL per well (2.5 μg/mL in Tris-HCl, pH 8.5) overnight at 4 °C. After supernatant removal and three PBST washes (PBS containing 0.5% Tween-20, pH 7.2–7.4), wells were blocked with 5% Difco skim milk at room temperature for 1 h. Subsequently, 100 μL of hybridoma supernatant or ascites-purified monoclonal antibodies were added to each well and incubated at 37 °C for 30 min. Following six PBST washes, a rabbit anti-mouse IgG-HRP conjugate (whole molecule, Sigma-Aldrich) diluted 1:2000 was applied and incubated for 30 min at 37 °C, followed by another six rounds of PBST washing. For color development, 100 μL/well of TMB substrate (TianGen, Beijing, China) was added, and plates were incubated for 20 min at room temperature in the dark. The reaction was terminated using 2 M H_2_SO_4_, and absorbance values at 450 nm were measured with an ELx808 Microplate Reader (BioTek, Shoreline, WA, USA).

### 2.6. IFA

Overexpressing cells were fixed in 4% paraformaldehyde and permeabilized with 0.1% Triton X-100 in PBS for 10 min to enable intracellular staining of both cytoplasmic and membrane-associated receptors. After three PBS washes, cells were blocked with 5% BSA for 1 h at 37 °C. Following three additional PBS rinses, cells were incubated with primary mAbs (diluted 1:2000) for 1 h at room temperature, followed by four washes with PBST. Samples were further incubated with the secondary antibody, goat anti-mouse IgG-Alexa Fluor 488 (Invitrogen, Waltham, MA, USA, 1:2000), for 1 h at 37 °C. Finally, fluorescent images were acquired using an inverted EVOS FL microscope (Life Technologies, Carlsbad, CA, USA).

### 2.7. Mouse Ascites Preparation and Monoclonal Antibody Purification

Hybridoma cells secreting specific monoclonal antibodies against mGluR2 were cultured to logarithmic growth phase. The cells were resuspended in serum-free RPMI-1640 medium at a density of 1 × 10^6^ cells/mL. Each mouse received an i.p. injection of 1.0 mL of the cell suspension. Ascites fluid was harvested from mice on days 7 to 14 following inoculation, followed by centrifugation at 12,000× *g* for 15 min at 4 °C to remove cellular debris and lipid aggregates. The clarified supernatant (containing the crude mAb) was carefully harvested, filtered through a 0.45 µm PES membrane (Millipore), and stored at −80 °C pending purification.

The monoclonal antibody was purified using a high-performance ÄKTA avant 150 system (Cytiva, Marlborough, MA, USA) equipped with a 1 mL HiTrap rProtein G FF pre-packed column (Cytiva). The column was initially equilibrated with 10 column volumes (CV) of binding buffer (20 mM sodium phosphate, 150 mM NaCl, pH 7.4) at a constant flow rate of 1 mL/min until a stable UV baseline was achieved. The pre-treated ascites sample was loaded onto the column at a flow rate of 1 mL/min. Following sample application, the column was washed with 10 CV of binding buffer at 1 mL/min to remove unbound host-derived proteins until the UV absorbance returned to baseline levels. The bound antibody was then eluted isocratically using 5 CV of elution buffer (0.1 M glycine-HCl, pH 2.7, supplemented with 150 mM NaCl). Fractions (0.5 mL) were automatically collected in 1.5 mL microcentrifuge tubes pre-filled with 50 µL of neutralization buffer (1.0 M Tris-HCl, pH 9.0) to instantaneously counteract the acidic pH.

### 2.8. Antibody Blocking Assay

To assess inhibition potential of mAbs to AIV infection, mAb solutions were prepared in Opti-MEM, with final concentrations of 30 μg/mL for the virus titration assay and 10 μg/mL and 50 μg/mL for the IFA. Isotype-matched IgG1 or IgG2a antibodies of the same concentration served as isotype controls. Confluent A549 monolayers were washed twice with ice-cold Opti-MEM and pre-cooled on ice for 10–15 min, and incubated with 40 μL of the respective mAb solutions per well at 4 °C for 1 h. Cells were then incubated with mixture of the same concentration of antibody and H9N2 virus (CK/GX/S11583-NA staygold/2019) at a multiplicity of infection (MOI) of 0.1 at 4 °C for 1 h. After incubation, the cells were washed three times with Opti-MEM containing the corresponding antibodies to remove unbound virus. Finally, 100 μL of mAb solution was added to each well, and the cells were incubated at 37 °C for 24 h. Culture supernatants were collected for viral titration, and virus-infected cells were observed under an inverted fluorescence microscope.

### 2.9. Monoclonal Antibody Sequencing

Total RNA was extracted from hybridoma cells, and cDNA was synthesized by reverse transcription using M-MLV Reverse Transcriptase (Invitrogen) and oligo (dT) primers. The major variable (V) and partial constant (C) regions of the heavy and light chains were amplified by multiple PCR runs and sequenced. The C-region gene fragment with the highest homology was screened against GenBank using Igblast (2023), and primers were designed to amplify the C region using PCR.

### 2.10. Statistical Analysis

All data were analyzed and graphed using GraphPad Prism (version 10.0, GraphPad Software, Boston, MA, USA). For ELISA binding assays, EC_50_ values were calculated by fitting the optical density values OD_450_ of serial antibody dilutions to a four-parameter logistic (4PL) non-linear regression model. Differences in antibody titers were analyzed using one-way analysis of variance (ANOVA). All viral neutralization data were derived from three independent biological replicates, each performed in triplicate technical replicates. All experiments were performed independently at least three times, and data are presented as mean ± standard deviation (mean ± SD). Statistical significance was determined using two-tailed tests, with *p* < 0.05 considered statistically significant. Significance levels are indicated as follows: * *p* < 0.05, ** *p* < 0.01, *** *p* < 0.001, and **** *p* < 0.0001.

## 3. Results

### 3.1. Expression, Purification, and Characterization of the Truncated mGluR2 Antigen

To generate highly specific mAbs against mGluR2, we engineered and expressed a recombinant truncated fragment of human mGluR2 in mammalian HEK293F cells. The expression of the target protein was first verified by Western blot analysis using an anti-His-tag antibody. As shown in Figure 1A and Figure S1, a specific immunoreactive band was observed at approximately 65 kDa in both the transfected HEK293F cell lysate (Lane 2) and the culture supernatant (Lane 3), whereas no signal was detected in the untransfected control cell lysate (Lane 1). This confirmed robust expression and secretion of the truncated mGluR2.

Subsequently, the secreted protein was captured from the culture supernatant using Ni-NTA affinity chromatography. SDS-PAGE analysis of the purification fractions revealed that the majority of the target protein bound efficiently to the His resin, with minimal protein remaining in the flow-through fraction (Lane 1 vs. Lane 2, Figure 1B). Eluted fractions yielded a highly enriched band of the expected molecular weight (Lane 3, Figure 1B). Following buffer exchange into PBS and subsequent concentration, the final protein product was analyzed by SDS-PAGE. The purified truncated mGluR2 exhibited a high degree of homogeneity and purity, appearing as a single, well-resolved band at approximately 65 kDa (Figure 1C). These results demonstrate that the recombinant antigen was successfully expressed and purified to a high level of purity, rendering it fully suitable for subsequent mouse immunization and monoclonal antibody screening.

### 3.2. mGluR2 MAb Preparation and Identification

To evaluate the humoral immune response induced by the purified truncated mGluR2 antigen, serum antibody titers from three immunized mice were measured using indirect ELISA. As shown in Figure 2A, all immunized mice exhibited robust antibody responses, with OD450 values remaining above the negative control level at serum dilutions up to 1:204,800. These results indicated successful and efficient immunogenic stimulation, promising high efficiency for subsequent cell fusion.

Following splenocyte fusion and consecutive rounds of limiting dilution subcloning, three specific mAb-producing hybridoma cell lines, designated as 1G3B2, 2D5A9, and 4E1C6, were successfully isolated. To evaluate the genetic and secretion stability of these hybridomas, the clones were continuously expanded and passaged up to 20 generations (P20). Indirect ELISA analysis revealed that the antigen-binding activity of the culture supernatants remained constant across different passages (Figure 2B). Furthermore, antibody isotyping analysis revealed that all three mAbs share an identical subclass composition, consisting of IgG1 heavy chains paired with Kappa light chains (Table 1). Taken together, these findings demonstrate that the three generated hybridoma clones are highly stable and capable of sustained production of mGluR2-specific monoclonal antibodies.

To further quantify the antigen-binding potency of the purified mAbs, dose–response binding curves were generated via indirect ELISA (Figure 2C). All three clones displayed typical sigmoidal binding kinetics toward mGluR2. Notably, 1G3B2 exhibited the highest binding affinity, yielding a half-maximal effective concentration (EC_50_) of 140.1 ng/mL. 2D5A9 and 4E1C6 displayed comparable binding profiles, with EC50 values of 175.3 ng/mL and 204.8 ng/mL, respectively. These quantitative binding parameters confirmed that all three mAbs exhibit strong apparent binding potency, with nanomolar-range apparent EC50 values suitable for downstream functional studies and immunoassays.

### 3.3. Functional Characterization and Application of the Screened Anti-mGluR2 mAbs

To evaluate the application potential and binding specificity of three mAbs, we performed Western blot and IFA to test their reactivity against denatured and native forms of mGluR2, respectively.

First, the efficacy of these mAbs in immunoblotting was evaluated using the purified truncated mGluR2 protein as the antigen. As shown in Figure 3A, all three isolated mAbs successfully recognized the denatured mGluR2 recombinant fragment, manifesting as clear and specific immunoreactive bands at approximately 65 kDa, consistent with the signal profile obtained from the positive mouse serum. In contrast, no detectable band was observed in the negative serum control. These results indicate that all three mAbs recognized the recombinant mGluR2 protein under denaturing conditions in Western blot.

To further determine whether these mAbs could recognize the native, full-length receptor expressed on mammalian cell membranes, an IFA was performed using HEK293T cells transiently transfected with full-length mGluR2. As illustrated in Figure 3B, strong green fluorescent signals were explicitly captured in cells incubated with the three mAbs (1G3B2, 2D5A9, and 4E1C6) as well as the positive serum control, showing characteristic membranous and cytoplasmic localization of the full-length receptor. No specific fluorescence was detected in the negative serum control group under the same imaging parameters. These findings demonstrate that the three generated mAbs not only bind efficiently to the linearized antigen but also showed reactivity with full-length mGluR2 overexpressed in HEK293T cells, showcasing their broad utility for both Western blot and IFA applications.

### 3.4. 1G3B2 as a Neutralizing Clone Against H9 Subtype Influenza Virus

To obtain high-yield monoclonal antibodies for functional blocking evaluation, the prepared hybridomas were inoculated into mice to generate immune ascites, followed by affinity purification. SDS-PAGE analysis confirmed the successful purification of three distinct mAb batches (Lanes 1–3), resolving as characteristic immunoglobulin heavy chains at approximately 50 kDa and light chains at approximately 25–27 kDa (Figure 4A).

We next investigated the potential of these purified mAbs to block H9 subtype AIV infection in vitro, utilizing a commercial murine anti-mGluR2 mAb and corresponding isotype matrices (IgG2a and IgG1) as controls. Interestingly, the viral replication profiles revealed a highly distinct epitope-dependent neutralizing capacity among the screened clones. As quantified by the viral titer assay (Figure 4B), 1G3B2 preincubation led to an approximately 7.7 folds (from 10^6.25^ to 10^5.36^ log10 TCID50/mL, corresponding to a 0.89 log decrease) reduction in viral titers compared to the controls, which closely mirrored the inhibitory phenotype of the commercial benchmarking anti-mGluR2 antibody. In contrast, clones 2D5A9 and 4E1C6 failed to exhibit viral blocking efficacy. These results suggest that while 2D5A9 and 4E1C6 are highly effective for diagnostic applications such as Western blot and IFA, only clone 1G3B2 targets an epitope associated with the inhibition of H9 virus infection.

To visually consolidate this neutralizing specificity, fluorescent signals was performed to track intracellular viral protein expression at two escalating concentrations (10 μg/mL and 50 μg/mL). In agreement with the viral titer dynamics, a discernible, dose-dependent trend in green fluorescent signals was observed qualitatively in 1G3B2-treated cells, particularly at the higher concentration of 50 μg/mL (Figure 4C). Conversely, cells treated with clones 2D5A9, 4E1C6, or the IgG1 isotype baseline sustained extensive, dense fluorescent signaling across both concentration cohorts. Taken together, these findings explicitly demonstrate that the prepared monoclonal antibody 1G3B2 exhibits inhibitory activity against H9N2 infection and can efficiently suppress H9 subtype influenza virus infection in vitro, suggesting a role in interfering with viral entry or replication, while the remaining two clones remain valuable candidates for alternative immunoassays.

### 3.5. Antibody Sequencing

To delineate the molecular basis of the neutralizing capacity of clone 1G3B2, the variable regions of its heavy VH and light VL chains were amplified and sequenced. Immunogenetic analysis aligned with the IMGT database revealed that the heavy chain variable region is rearranged from the germline genes IGHV5-6-2*01, IGHD4-1*01, and IGHJ3*01, with its constant region determined by IGHG101_BALB/c. For the light chain, the variable region is derived from the germline gene recombination of IGKV3-4*01 and IGKJ1*01, paired with the IGKC*01_musculus constant region. Furthermore, the precise amino acid sequences of the complementary determining regions (CDRs), which dictate antigen recognition, were characterized (Table 2). The VH domain consists of a 296 bp nucleotide sequence, encoding CDR1 (GFTFSNYF), CDR2 (INSNGGMT), and a relatively prolonged CDR3 loop (TRHPVNNWAWFAY). The VL domain, spanning 296 bp, comprises CDR1 (QSVDYDGDSY), CDR2 (VAS), and CDR3 (LQSNEDPWT). The sequence determination of the CDR loops offers invaluable primary structure data.

## 4. Discussion

In this study, we successfully generated and characterized a panel of monoclonal antibodies (mAbs) targeting mGluR2, a critical endocytic receptor for influenza virus. The three stable hybridoma clones, 1G3B2, 2D5A9, and 4E1C6, exhibited high binding affinity to both denatured and native forms of mGluR2. Notably, clone 1G3B2 demonstrated potent neutralizing activity against the H9 subtype influenza virus, significantly reducing viral titers by approximately 7.7 folds. In contrast, clones 2D5A9 and 4E1C6 showed no viral blocking efficacy, highlighting their utility for diagnostic applications. Additionally, we resolved the genetic characteristics of the heavy and light chain variable regions of 1G3B2, providing a foundation for future structural and translational studies.

The functional heterogeneity observed among the three mAbs underscores the importance of epitope specificity in determining their biological activity. All three mAbs bound efficiently to the truncated mGluR2 protein and recognized the full-length receptor on eukaryotic cell membranes. However, only 1G3B2 significantly reduced viral titers in neutralization assays. This phenomenon, where an antibody binds to an antigen but fails to block its function, is not uncommon in research targeting host receptors [20,21]. As a seven-transmembrane G-protein-coupled receptor, mGluR2′s extracellular region contains a Venus flytrap domain and a cysteine-rich domain. The epitopes recognized by 2D5A9 and 4E1C6 may reside in peripheral, non-functional regions of mGluR2, thus failing to exert steric hindrance on viral HA binding or the conformational changes required for clathrin-coated pit maturation [6,22]. Conversely, the epitope targeted by 1G3B2 likely overlaps with the HA-binding region or a critical domain involved in mGluR2′s functional conformational changes, although the precise mechanistic step remains to be determined. These results indicate that 1G3B2 is not merely an immunological tool but an effective molecular blocker probably by disrupting receptor-mediated endocytosis. Meanwhile, 2D5A9 and 4E1C6, due to their excellent binding affinity, remain valuable as research reagents for applications such as Western blot and immunofluorescence assays.

Although 1G3B2 strongly inhibited viral titers, fluorescence signals revealed a dose-dependent decrease rather than complete elimination of intracellular viral antigen signals. This highlights the complex entry dynamics of the influenza virus into host cells [23,24]. The endocytic process involves an initial sialic acid-mediated attachment followed by the cooperative involvement of a multi-receptor network, including EGFR and mGluR2. Under conditions with a high multiplicity of infection (MOI), blocking the mGluR2 pathway alone can significantly reduce endocytic efficiency and progeny viral replication but may not completely shut down alternative entry routes [11,24]. This finding suggests a direction for future functional studies, where 1G3B2 could be combined with targeted drugs against other host entry factors (such as EGFR blockers) or neuraminidase inhibitors to explore multi-target synergistic strategies for complete viral entry blockade.

We also performed gene sequencing and germline analysis on the variable regions of the neutralizing mAb 1G3B2, obtaining the amino acid sequences of its variable domains. The heavy chain variable region (V_H_, 296 bp) is rearranged from the BALB/c germline genes IGHV5-6-2*01/IGHD4-1*01/IGHJ3*01, and the light chain variable region (V_L_, 296 bp) is recombined from IGKV3-4*01/IGKJ1*01 and paired with IGKC*01. The successful sequencing of these regions permanently preserves the molecular information of 1G3B2, establishing a solid foundation for standardized, animal-component-free, large-scale recombinant antibody production using eukaryotic expression systems such as CHO or HEK293 cells [25]. The unique CDR3 sequence of 1G3B2, particularly the prolonged heavy chain CDR3 loop (TRHPVNNWAWFAY), provides a sequence baseline for future structural biology studies using cryo-electron microscopy (cryo-EM) or X-ray crystallography to resolve the mGluR2-1G3B2 complex structure. This will help elucidate the exact neutralization mechanism at an atomic level. Furthermore, this sequence information enables the humanization of the antibody framework regions through genetic engineering methods like CDR grafting, minimizing the risk of immunogenicity in clinical applications [26].

Despite the robust findings, our study has limitations. First, the in vitro neutralization assays were conducted with a single H9 subtype influenza virus, and the broad-spectrum efficacy of 1G3B2 against other subtypes remains to be evaluated. Second, the long-term stability and potential immunogenicity of 1G3B2 in vivo need further investigation. Future studies should address these aspects to fully realize the therapeutic potential of 1G3B2.

## 5. Conclusions

This study provides a neutralizing antibody candidate that suppresses H9N2 replication in vitro. The distinct functional profiles of the generated mAbs highlight the importance of epitope specificity in antiviral interventions, while the genetic characterization of 1G3B2 paves the way for advanced structural and translational research in the future.

## Figures and Tables

**Figure 1:**
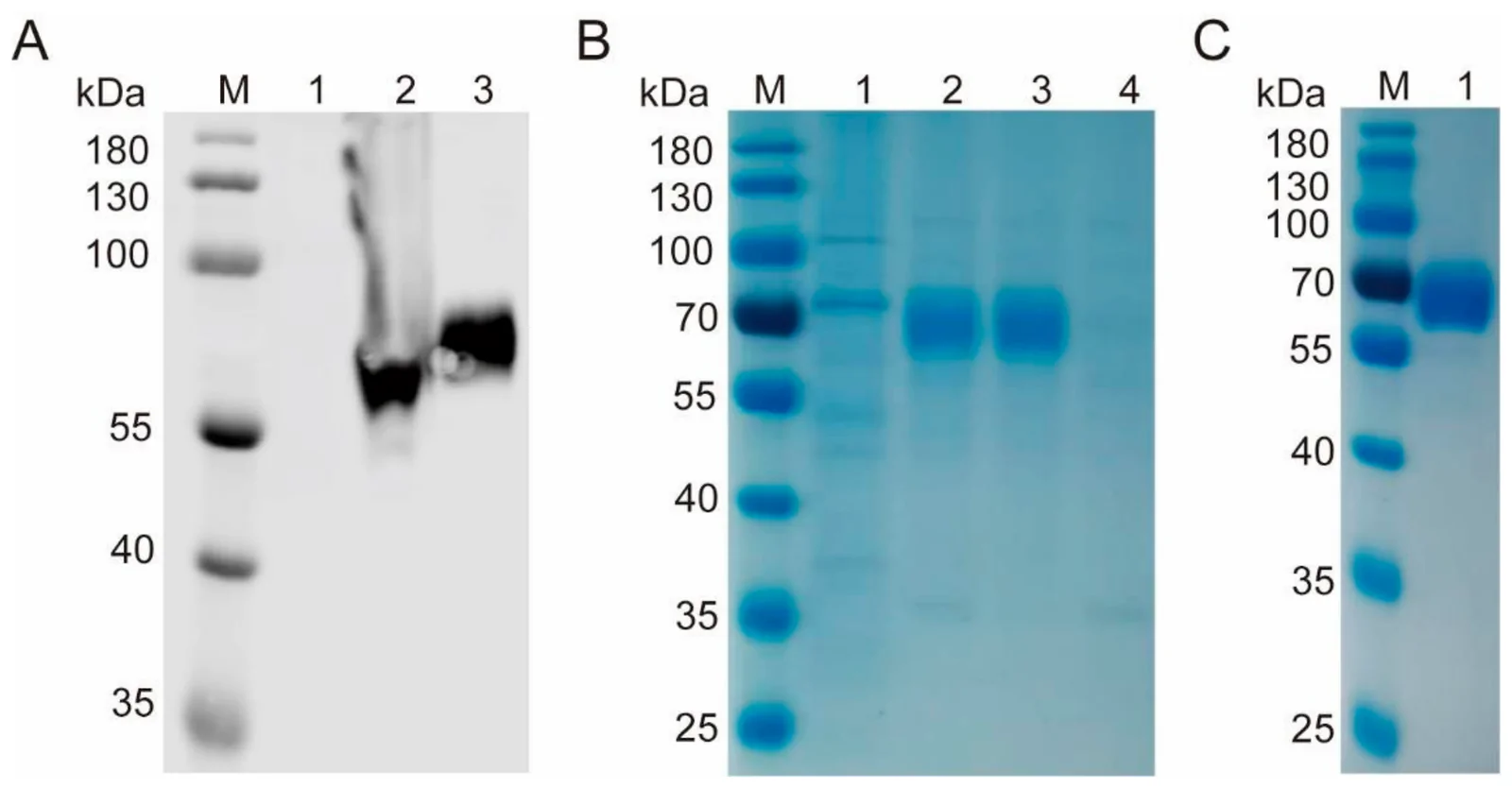
Expression and purification of truncated mGluR2 protein. (**A**) Western blot analysis of the truncated mGluR2 expression in HEK293F cells. M, Protein molecular weight marker; 1, Untransfected HEK293F cell lysate (negative control); 2, Transfected HEK293F cell lysate; 3, Culture supernatant. (**B**) SDS-PAGE profile of the Ni-NTA affinity purification. M, Protein molecular weight marker; 1, Unbound flow-through (supernatant); 2, Target protein bound to His resin; 3, Eluted truncated mGluR2 fraction; 4, Resin profile post-elution. (**C**) SDS-PAGE identification of the purified protein. M, Protein molecular weight marker; 1, Purified truncated mGluR2.

**Figure 2:**
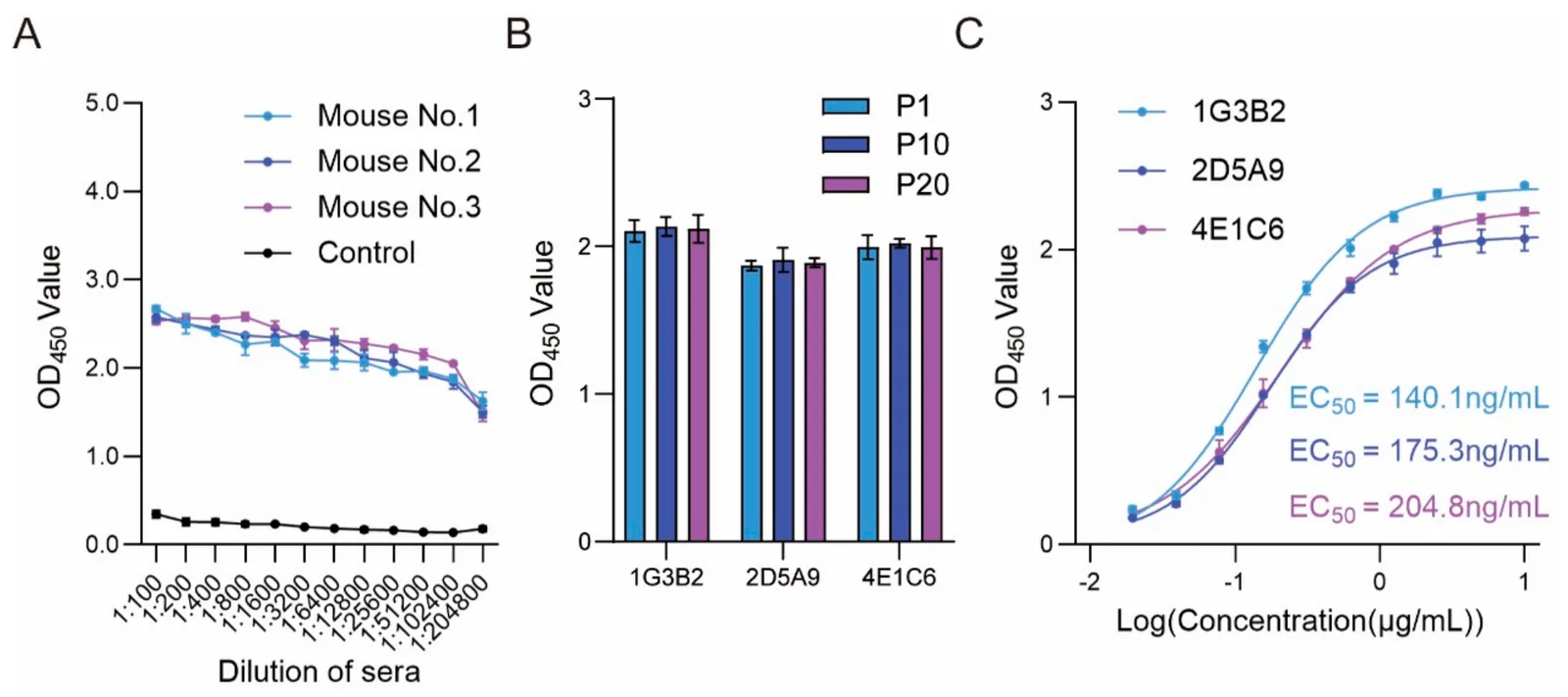
Screening and identification of anti-mGluR2 mAbs. (**A**) Determination of serum antibody titers in mGluR2-immunized mice prior to splenocyte fusion by indirect ELISA. Serum samples from three immunized mice (Mice No. 1−3) and an unimmunized negative mouse were serially diluted from 1:100 to 1:204,800. Data are presented as mean ± SD. (**B**) Stability assessment of antibody secretion by the isolated hybridoma clones across successive passages. The culture supernatants of the three selected clones (1G3B2, 2D5A9, and 4E1C6) at passage 1 (P1), passage 10 (P10), and passage 20 (P20) were collected and analyzed by indirect ELISA against the mGluR2 antigen. Data are shown as mean ± SD. (**C**) Dose-dependent binding curves and EC_50_ determination of purified mAbs against recombinant mGluR2. Log-transformed antibody concentrations (μg/mL) are plotted against OD_450_ values. Data represent mean ± SD from three independent replicates.

**Figure 3:**
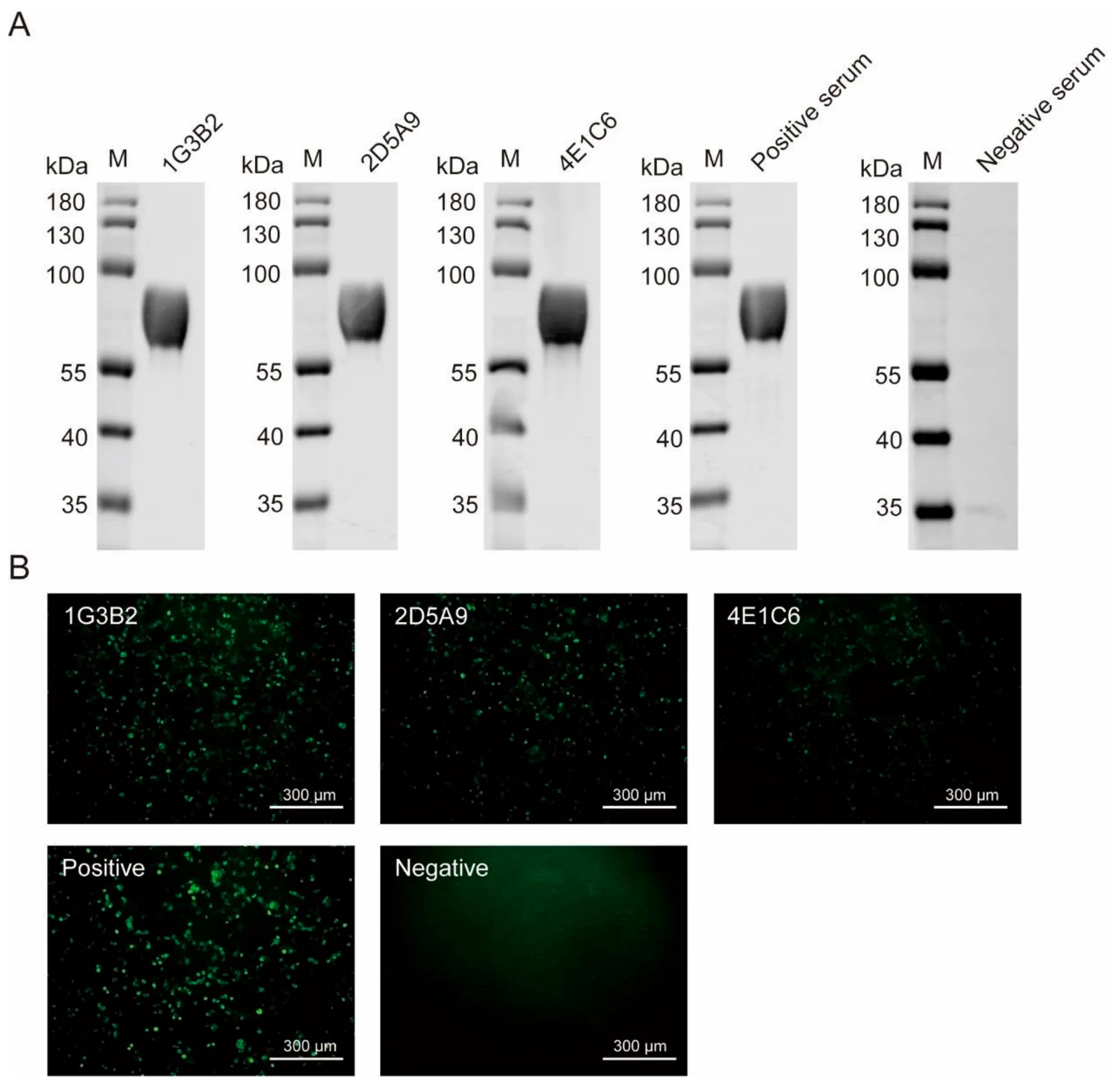
Validation of anti-mGluR2 mAbs in Western blot and immunofluorescence assays. (**A**) Specificity validation of the three mAbs by Western blot using the purified truncated mGluR2 antigen. Anti-mGluR2 mouse serum (Positive) and negative mouse serum (Negative) were used as controls. (**B**) IFA of the three mAbs against HEK293T cells transiently transfected with full-length mGluR2. Green fluorescence represents specific binding of the mAbs or serum controls to the full-length receptor.

**Figure 4:**
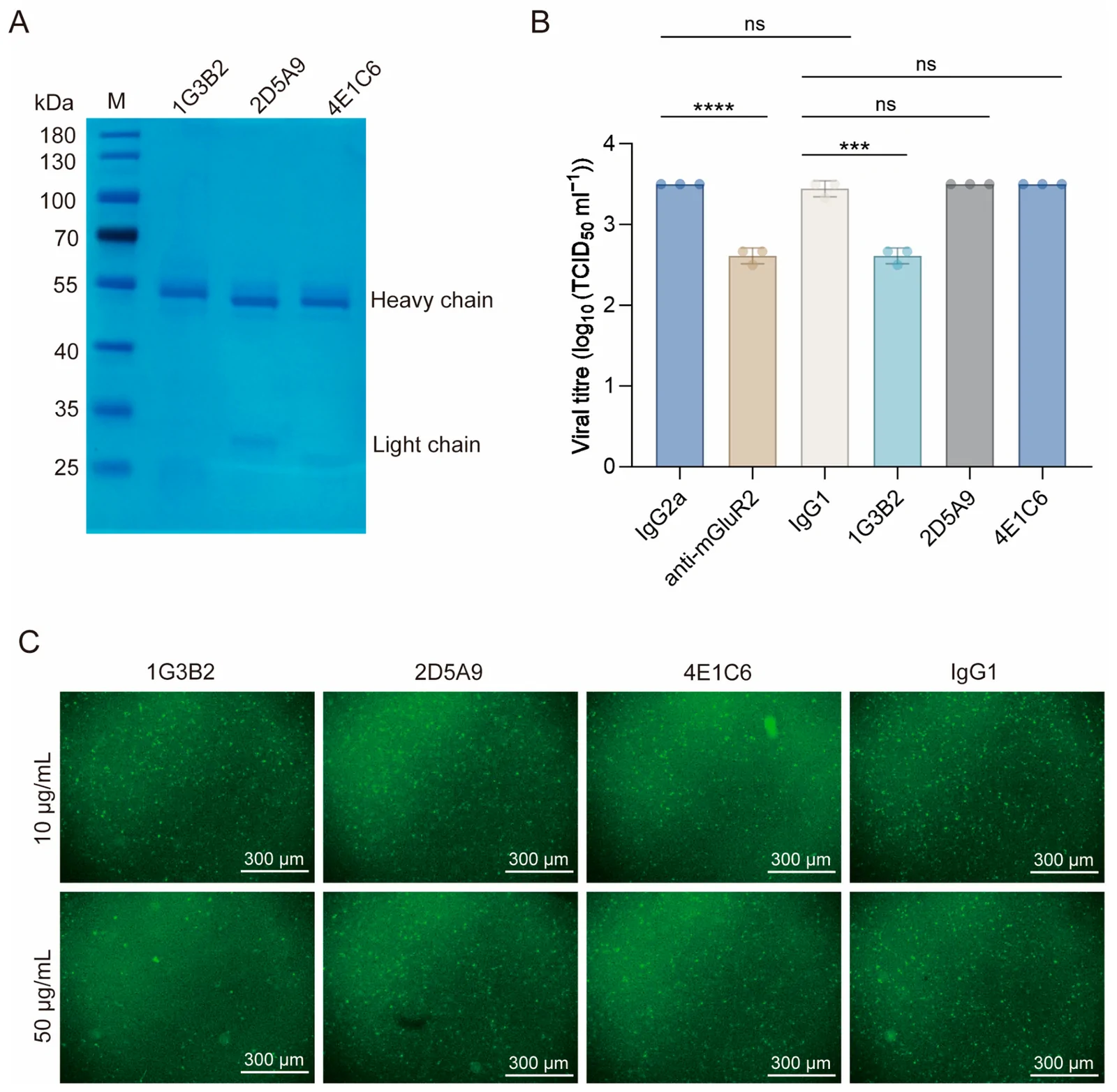
Inhibition capacities of mAbs against H9 subtype influenza virus. (**A**) SDS-PAGE characterization of the three purified anti-mGluR2 mAb batches from mouse ascites. M, Molecular weight marker; 1−3: Purified mAb clones corresponding to 1G3B2, 2D5A9, and 4E1C6, respectively. (**B**) In vitro receptor-blocking evaluation quantified by viral titration. Host cells were pre-incubated with the mAbs (1G3B2, 2D5A9, 4E1C6), a commercial anti-mGluR2 mAb, or their respective isotype controls (IgG2a or IgG1) for 1 h at 4 °C, the infected with 0.1 MOI of H9N2 virus (CK/GX/S11583-NA staygold/2019). Viral titers were assessed 24 h post infection and measured as log_10_(TCID_50_/mL). Data are presented as mean ± SD. Statistical significance was determined using one-way ANOVA followed by Dunnett’s multiple comparisons test against the IgG1 isotype control group. *** *p* < 0.001, **** *p* < 0.0001; ns, not significant (*p* > 0.05). (**C**) H9N2 viral protein expression in host cells was visualized using fluorescence signals under antibody blocking constraints. Cells were pre-incubated with either (10 μg/mL and 50 μg/mL) of the specified mAbs or the IgG1 isotype control for 1 h at 4 °C, the infected with 0.1 MOI of H9N2 virus (CK/GX/S11583-NA staygold/2019). Green puncta denote intracellular viral proteins. Data represent mean ± SD from three independent experiments (n = 3 biological replicates, each with 3 technical replicates).

**Table 1:** Identification results of the mAbs subtypes.

Monoclonal Antibodies	Subclass and Types (OD_450nm_)							
	IgG 1	IgG 2a	IgG 2b	IgG 3	IgG A	IgG M	Kappa	Lambda
1G3B2	2.332	0.387	0.214	0.185	0.263	0.091	1.658	0.076
2D5A9	1.983	0.264	0.188	0.094	0.168	0.087	1.403	0.052
4E1C6	1.876	0.179	0.162	0.231	0.247	0.103	1.582	0.084

Note: Purple background indicates positive results.

**Table 2:** CDR Sequences of mAb 1G3B2.

mAb	Chain	Length ^a^	CDR1	CDR2	CDR3
1G3B2	H	296 bp	GFTFSNYF	INSNGGMT	TRHPVNNWAWFAY
	L	296 bp	QSVDYDGDSY	VAS	LQSNEDPWT

^a^: V gene length of the heavy or light chains.

## Data Availability

The original contributions presented in this study are included in the article/Supplementary Materials. Further inquiries can be directed to the corresponding author.

## Supplementary Materials

The following supporting information can be downloaded at https://www.mdpi.com/article/10.3390/vetsci13101001/s1. Figure S1: Original images of Western-blotting.
