# Supplementary material for: A Monoclonal Antibody Targeting mGluR2 Inhibits H9 Subtype Influenza A Virus Infection In Vitro
Source: Vet Sci. 2026 Sep 23;13(10):1001. doi: 10.3390/vetsci13101001 (PMC13709593; doi:10.3390/vetsci13101001)
Supplement: Supplementary file 1 [file vetsci-13-01001-s001.zip › Figure S1 Original Images of WB.pdf]

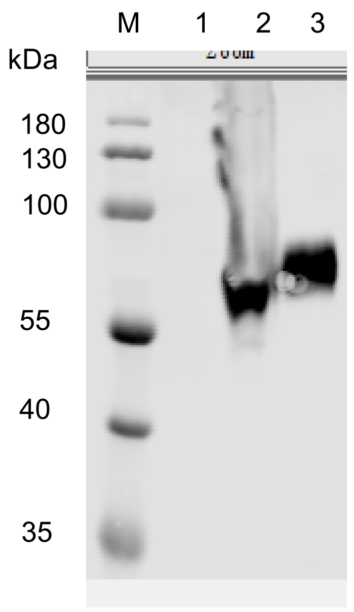

Fig. 1 A

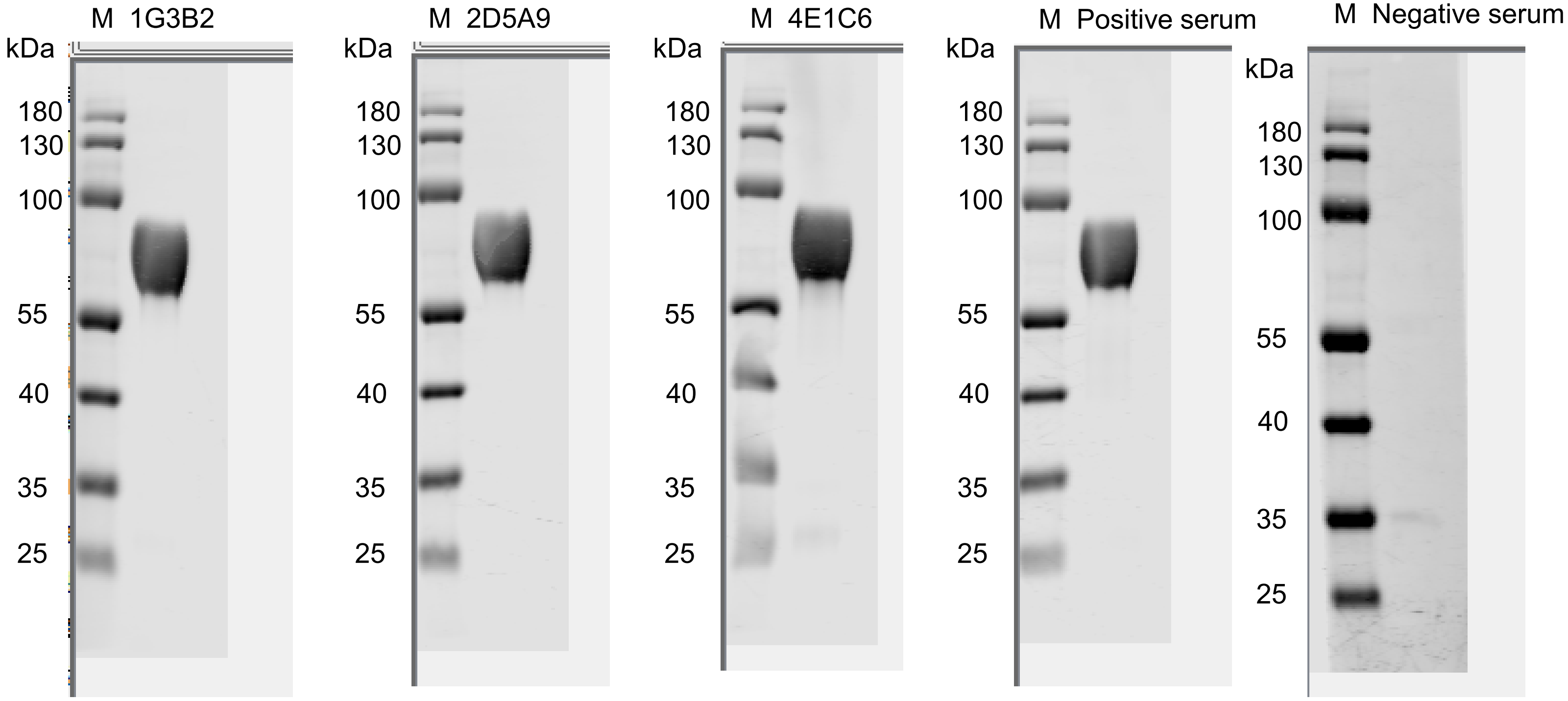
M 1G3B2
M 2D5A9
M 4E1C6
M Positive serum
M Negative serum
kDa
180
130
100
55
40
35
25

Fig. 3 A
